# Graham Bread

**Published:** 1875-08

**Authors:** 


### GRAHAM BREAD.

Sylvester Graham introduced the idea of employing the coarse part of ground wheat in the manufacture of bread, puddings and the like. It has been said that he starved himself to death by attempting to live on bran-food. This is untrue. "Graham" was, however, long a term of reproach as applied to food. But it has weathered the storm, and now no bake-shop or bill of fare is complete without "Graham bread.

But the bread now called Graham is usually poor trash. It is either what remains after the fine and white portions have been sifted out, or it is bad flour mixed with bran or "shorts." Neither bran nor the mixture used by the bakers is fit for human beings to eat. Probably good superfine flour uncontaminated by acids and alkalis and grease, is better than either of them, if baked to brittleness and softened only by mastication and saliva. Yet superfine flour is not the best food, by any means. No bread can be made as wholesome, as digestable and as powerful in building up, as that made from choice grains, ground, unsifted, mixed only with water and baked to brownness. This is the ideal "Graham," and is vastly superior to any other bread known. It matters little which grain is used, so that it is sweet and perfect.

Flour manufacturers know that "anything will do for Graham." Sour grain, diseased grain, the sweepings of the mill, dirt, anything in short will answer the purpose of the national creatures who cry for Graham meal. So the waste, the wretched refuse which can't be sold for any other purpose save for horse-feed, is worked off for the use of dyspeptics, and they congratulate themselves on their delightful unbolted wheat bread.

All this is rather severe on the poor sufferers who are seeking to live wisely and who know that it is not wise to sift from the ground grain some of the most valuable elements. But for a great majority there is no relief. Those who have the time and the ability, can buy choice grain and have it ground by some good miller; those who cannot do this must suffer the consequences, But let none of our readers suppose for a moment that the Graham loaf which they buy from the baker is any better than any other baker's loaf, if as good. It is merely his dirtiest product and his worst. It is made from cheap flour and a sprinkling of bran or shorts, just enough to give it a coarse look. It is villainous trash. Besides, it is usually sweetened and is thus rendered more abominable still.

Absolutely, we know of only one bread in the market which exactly meets the wants of those who desire unbolted wheat food in perfection. This is the "Granulated Wheat Biscuit," made from pounded grain and water only, mixed by machinery and baked in soap-stone ovens. We describe the process fully elsewhere. It is an admirable food and is being eagerly sought for by sensible people everywhere.

---

To Mend and Clean Kid Gloves.—Turn them on the wrong side, and sew them over and over in the ordinary way. They will last longer and look better, if mended on the wrong side. Turn them back again, and go over them with a clean towel dipped in skim milk and rubbed lightly on white castile soap, wearing them during the process and until they are quite dry.

---

To Restore Color.—When color on a fabric has been accidentally or otherwise destroyed by acid, ammonia is applied to neutralize the acid, after which an application of chloroform will, in almost all cases, restore the original color. The application of ammonia is common, but that of chloroform is but little known.

---

A grand necessity elevates a man; a small one degrades him.

